# Supplementary material for: White matter hyperintensities classified according to intensity and spatial location reveal specific associations with cognitive performance
Source: Neuroimage Clin. 2021 Mar 7;30:102616. doi: 10.1016/j.nicl.2021.102616 (PMC7995650; doi:10.1016/j.nicl.2021.102616)
Supplement: Supplementary data 1 [file mmc1.docx]

**Supplementary Material**

**White matter hyperintensities classified according to intensity and spatial location reveal specific associations with cognitive performance**

Luca Melazzini^a,b^, Clare E Mackay^b,c,d^, Valentina Bordin^b,e^, Sana Suri^c,f^, Enikő Zsoldos^b,c,f^, Nicola Filippini^c^, Abda Mahmood^f^, Vaanathi Sundaresan^b^, Marina Codari^g^, Eugene Duff^b,h^, Archana Singh-Manoux^i,j^, Mika Kivimäki^j^, Klaus P Ebmeier^f^, Mark Jenkinson^b^, Francesco Sardanelli^a,k^, Ludovica Griffanti^b,c^.

^a^ Department of Biomedical Sciences for Health, Università degli Studi di Milano, Milan, Italy

^b^ Wellcome Centre for Integrative Neuroimaging, Oxford Centre for Functional MRI of the Brain, Nuffield Department of Clinical Neurosciences, University of Oxford, Oxford, UK

^c^ Wellcome Centre for Integrative Neuroimaging, Oxford Centre for Human Brain Activity, Department of Psychiatry, University of Oxford, Oxford, UK

^d^ Oxford Health NHS Foundation Trust, Oxford, UK

^e^ Department of Electronics, Information and Bioengineering, Politecnico di Milano, Milan, Italy

^f^ Department of Psychiatry, Warneford Hospital, University of Oxford, Oxford, UK

^g^ Department of Radiology, Stanford University School of Medicine, Stanford, USA

^h^ Department of Paediatrics, University of Oxford, Oxford, UK

^i^ INSERM U1153, Epidemiology of Ageing and Neurodegenerative diseases, Université de Paris, Paris, France.

^j^ Department of Epidemiology and Public Health, University College London, London, UK

^k^ Department of Radiology, IRCCS Policlinico San Donato, Milan, Italy

**Supplementary Tables**

Table S1. Acquisition parameters of T_1_-weighted, FLAIR and DWI images on Siemens 3-T Verio and Prisma scanners.

| Sequence | T_1_-weighted | | FLAIR | | DWI | |
| --- | --- | --- | --- | --- | --- | --- |
| Scanner | Verio | Prisma | Verio | Prisma | Verio | Prisma |
| TR, *ms* | 2530 | 1900 | 9000 | 9000 | 8900 | 8900 |
| TE, *ms* | 1.79 / 3.65 / 5.51 / 7.37 | 3.97 | 73 | 73 | 91.2 | 91 |
| Flip angle | 7° | 8° | 150° | 150° | ----- | ----- |
| Voxel, *mm^3^* | 1 x 1 x 1 | 1 x 1 x 1 | 0.9 x 0.9 x 3 | 0.4 x 0.4 x 3 | 2 x 2 x 2 | 2 x 2 x 2 |
| FoV read | 256 | 192 | 220 | 220 | 192 | 192 |
| FoV phase | 100% | 100% | 100% | 100% | 100% | 100% |
| Base resolution | 256 | 192 | 256 | 256 | 96 | 96 |
| Phase resolution | 100% | 100% | 100% | 100% | 100% | 100% |
| TI, *ms* | 1380 | 904 | 2500 | 2500 | ----- | ----- |
| Bandwith, *Hz/Px* | 651 | 200 | 283 | 283 | 1680 | 1680 |
| Orientation | Sagittal | Transversal | Transversal | Transversal | Transversal | Transversal |
| b-value, *s/mm^2^* | ----- | ----- | ----- | ----- | 1500 | 1500 |
| Directions, *N* | ----- | ----- | ----- | ----- | 60 + 5 b=0 | 60 + 5 b=0 |
| Acquisition time | 6'12" | 5'31" | 4'14" | 4'14" | 10'32" | 10'41" |

Legend: FLAIR, fluid attenuated inversion recovery; DWI, diffusion weighted imaging; TR, repetition time; TE, echo time; FoV, field of view; TI, inversion time, Acquisition T; acquisition time.

Table S2. Multiple linear regression analysis comprehensive results. Sub-classes of WMH that significantly predict cognitive scores are reported in bold.

|  | Model | | | Parameter estimates | | |
| --- | --- | --- | --- | --- | --- | --- |
| Cognitive test | F | p-value | Adjusted R^2^ | Significant covariate | β (SE) | p-value |
|  |  |  |  |  |  |  |
| MoCA | 5.986 | <0.001 | 0.062 | Age | -0.119 (0.019) | <0.001 |
|  |  |  |  | Sex (female) | -0.020 (0.217) | 0.927 |
|  |  |  |  | Systolic blood pressure | 0.000 (0.007) | 0.976 |
|  |  |  |  | Diastolic blood pressure | -0.003 (0.010) | 0.792 |
|  |  |  |  | Education | -0.007 (0.030) | 0.806 |
|  |  |  |  | T_1_-hypointense periventricular WMHs | -0.082 (0.366) | 0.822 |
|  |  |  |  | Non T_1_-hypointense periventricular WMHs | 0.079 (0.519) | 0.879 |
|  |  |  |  | T_1_-hypointense deep WMHs | -0.014 (0.302) | 0.962 |
|  |  |  |  | Non T_1_-hypointense deep WMHs | 0.302 (0.358) | 0.399 |
|  |  |  |  |  |  |  |
| **Trail making A** | 10.082 | <0.001 | 0.108 | Age | 0.615 (0.097) | <0.001 |
|  |  |  |  | Sex (female) | 1.102 (1.114) | 0.323 |
|  |  |  |  | Systolic blood pressure | 0.002 | 0.958 |
|  |  |  |  | Diastolic blood pressure | 0.009 | 0.864 |
|  |  |  |  | Education | -0.342 (0.153) | 0.025 |
|  |  |  |  | **T_1_-hypointense periventricular WMHs** | **4.792 (1.870)** | **0.011** |
|  |  |  |  | Non T_1_-hypointense periventricular WMHs | -3.328 | 0.204 |
|  |  |  |  | T_1_-hypointense deep WMHs | 0.495 | 0.749 |
|  |  |  |  | Non T_1_-hypointense deep WMHs | -1.502 | 0.412 |
|  |  |  |  |  |  |  |
| Trail making B | 10.374 | <0.001 | 0.111 | Age | 1.556 (0.277) | <0.001 |
|  |  |  |  | Sex (female) | 7.387 (3.182) | 0.021 |
|  |  |  |  | Systolic blood pressure | 0.198 (0.095) | 0.038 |
|  |  |  |  | Diastolic blood pressure | -0.141 (0.153) | 0.358 |
|  |  |  |  | Education | -1.316 (0.434) | 0.003 |
|  |  |  |  | T_1_-hypointense periventricular WMHs | 9.637 (5.324) | 0.071 |
|  |  |  |  | Non T_1_-hypointense periventricular WMHs | -8.192 (7.566) | 0.279 |
|  |  |  |  | T_1_-hypointense deep WMHs | 0.106 (4.406) | 0.981 |
|  |  |  |  | Non T_1_-hypointense deep WMHs | -2.233 (5.219) | 0.669 |
|  |  |  |  |  |  |  |
| Trail making B-A | 6.653 | <0.001 | 0.070 | Age | 0.962 (0.240) | <0.001 |
|  |  |  |  | Sex (female) | 7.073 (2.759) | 0.011 |
|  |  |  |  | Systolic blood pressure | 0.187 (0.082) | 0.024 |
|  |  |  |  | Diastolic blood pressure | -0.128 (0.132) | 0.336 |
|  |  |  |  | Education | -0.898 (0.376) | 0.017 |
|  |  |  |  | T_1_-hypointense periventricular WMHs | 5.901 (4.617) | 0.202 |
|  |  |  |  | Non T_1_-hypointense periventricular WMHs | -4.977 (6.561) | 0.448 |
|  |  |  |  | T_1_-hypointense deep WMHs | 0.288 (3.821) | 0.940 |
|  |  |  |  | Non T_1_-hypointense deep WMHs | -1.996 (4.525) | 0.659 |
|  |  |  |  |  |  |  |
| Digit span forward | 6.539 | <0.001 | 0.068 | Age | -0.045 (0.019) | 0.019 |
|  |  |  |  | Sex (female) | -0.571 (0.221) | 0.010 |
|  |  |  |  | Systolic blood pressure | 0.000 (0.007) | 0.954 |
|  |  |  |  | Diastolic blood pressure | -0.003 (0.011) | 0.803 |
|  |  |  |  | Education | 0.144 (0.030) | <0.001 |
|  |  |  |  | T_1_-hypointense periventricular WMHs | -0.681 (0.371) | 0.067 |
|  |  |  |  | Non T_1_-hypointense periventricular WMHs | -0.254 (0.527) | 0.629 |
|  |  |  |  | T_1_-hypointense deep WMHs | 0.207 (0.307) | 0.500 |
|  |  |  |  | Non T_1_-hypointense deep WMHs | 0.384 (0.363) | 0.290 |
|  |  |  |  |  |  |  |
| **Digit span backwards** | 5.917 | <0.001 | 0.061 | Age | -0.072 (0.021) | 0.001 |
|  |  |  |  | Sex (female) | -0.419 (0.239) | 0.080 |
|  |  |  |  | Systolic blood pressure | 0.008 (0.007) | 0.290 |
|  |  |  |  | Diastolic blood pressure | 0.001 (0.012) | 0.920 |
|  |  |  |  | Education | 0.137 (0.033) | <0.001 |
|  |  |  |  | T_1_-hypointense periventricular WMHs | -0.577 (0.402) | 0.151 |
|  |  |  |  | **Non T_1_-hypointense periventricular WMHs** | **1.300 (0.571)** | **0.023** |
|  |  |  |  | T_1_-hypointense deep WMHs | -0.116 (0.332) | 0.726 |
|  |  |  |  | Non T_1_-hypointense deep WMHs | 0.222 (0.393) | 0.571 |
|  |  |  |  |  |  |  |
| Digit span sequence | 4.988 | <0.001 | 0.050 | Age | -0.086 (0.021) | <0.001 |
|  |  |  |  | Sex (female) | -0.379 (0.243) | 0.119 |
|  |  |  |  | Systolic blood pressure | 0.005 (0.007) | 0.501 |
|  |  |  |  | Diastolic blood pressure | -0.005 (0.012) | 0.668 |
|  |  |  |  | Education | 0.067 (0.033) | 0.044 |
|  |  |  |  | T_1_-hypointense periventricular WMHs | -0.787 (0.408) | 0.054^ |
|  |  |  |  | Non T_1_-hypointense periventricular WMHs | 0.206 (0.580) | 0.723 |
|  |  |  |  | T_1_-hypointense deep WMHs | 0.420 (0.337) | 0.214 |
|  |  |  |  | Non T_1_-hypointense deep WMHs | -0.391 (0.399) | 0.327 |
|  |  |  |  |  |  |  |
| **Digit symbol** | 8.249 | <0.001 | 0.088 | Age | -0.202 (0.048) | <0.001 |
|  |  |  |  | Sex (female) | -1.369 (0.551) | 0.013 |
|  |  |  |  | Systolic blood pressure | 0.013 (0.017) | 0.437 |
|  |  |  |  | Diastolic blood pressure | -0.007 (0.027) | 0.807 |
|  |  |  |  | Education | 0.348 (0.075) | <0.001 |
|  |  |  |  | **T_1_-hypointense periventricular WMHs** | **-2.045 (0.927)** | **0.028** |
|  |  |  |  | Non T_1_-hypointense periventricular WMHs | 1.251 (1.317) | 0.342 |
|  |  |  |  | T_1_-hypointense deep WMHs | 0.511 (0.766) | 0.505 |
|  |  |  |  | Non T_1_-hypointense deep WMHs | 0.216 (0.906) | 0.812 |
|  |  |  |  |  |  |  |
| **Digit coding** | 11.062 | <0.001 | 0.118 | Age | -0.606 (0.109) | <0.001 |
|  |  |  |  | Sex (female) | 1.593 (1.242) | 0.200 |
|  |  |  |  | Systolic blood pressure | -0.052 (0.037) | 0.161 |
|  |  |  |  | Diastolic blood pressure | 0.082 (0.060) | 0.174 |
|  |  |  |  | Education | 0.618 (0.171) | <0.001 |
|  |  |  |  | **T_1_-hypointense periventricular WMHs** | **-5.524 (2.093)** | **0.009** |
|  |  |  |  | Non T_1_-hypointense periventricular WMHs | 4.883 (2.976) | 0.101 |
|  |  |  |  | T_1_-hypointense deep WMHs | -1.950 (1.732) | 0.260 |
|  |  |  |  | Non T_1_-hypointense deep WMHs | 1.997 (2.049) | 0.330 |
|  |  |  |  |  |  |  |
| Boston naming-60 | 5.926 | <0.001 | 0.061 | Age | -0.063 (0.040) | 0.114 |
|  |  |  |  | Sex (female) | -2.393 (0.454) | <0.001 |
|  |  |  |  | Systolic blood pressure | -0.028 (0.014) | 0.041 |
|  |  |  |  | Diastolic blood pressure | 0.041 (0.022) | 0.064 |
|  |  |  |  | Education | 0.150 (0.062) | 0.016 |
|  |  |  |  | T_1_-hypointense periventricular WMHs | 0.312 (0.764) | 0.684 |
|  |  |  |  | Non T_1_-hypointense periventricular WMHs | 0.849 (1.085) | 0.434 |
|  |  |  |  | T_1_-hypointense deep WMHs | -0.299 (0.632) | 0.637 |
|  |  |  |  | Non T_1_-hypointense deep WMHs | 0.408 (0.747) | 0.585 |
|  |  |  |  |  |  |  |
| **Letter fluency** | 3.737 | <0.001 | 0.035 | Age | -0.077 (0.038) | 0.046 |
|  |  |  |  | Sex (female) | -0.118 (0.439) | 0.788 |
|  |  |  |  | Systolic blood pressure | -0.008 (0.013) | 0.545 |
|  |  |  |  | Diastolic blood pressure | 0.022 (0.021) | 0.294 |
|  |  |  |  | Education | 0.154 (0.060) | 0.011 |
|  |  |  |  | T_1_-hypointense periventricular WMHs | -1.069 (0.740) | 0.149 |
|  |  |  |  | Non T_1_-hypointense periventricular WMHs | -0.051 (1.051) | 0.962 |
|  |  |  |  | T_1_-hypointense deep WMHs | -1.137 (0.612) | 0.063 |
|  |  |  |  | **Non T_1_-hypointense deep WMHs** | **2.097 (0.723)** | **0.004** |
|  |  |  |  |  |  |  |
| **Category fluency** | 8.071 | <0.001 | 0.086 | Age | -0.242 (0.046) | <0.001 |
|  |  |  |  | Sex (female) | -0.330 (0.529) | 0.533 |
|  |  |  |  | Systolic blood pressure | -0.001 (0.016) | 0.948 |
|  |  |  |  | Diastolic blood pressure | 0.028 (0.026) | 0.275 |
|  |  |  |  | Education | 0.295 (0.073) | <0.001 |
|  |  |  |  | T_1_-hypointense periventricular WMHs | -0.658 (0.891) | 0.460 |
|  |  |  |  | Non T_1_-hypointense periventricular WMHs | 0.504 (1.265) | 0.690 |
|  |  |  |  | T_1_-hypointense deep WMHs | -0.852 (0.737) | 0.248 |
|  |  |  |  | **Non T_1_-hypointense deep WMHs** | **1.829 (0.871)** | **0.036** |

Legend: MoCA, Montreal cognitive assessment; SE, standard error; WMHs, white matter hyperintensities.
^Predictor that shows a trend for significance (0.05<p<0.06).

Table S3. Multiple linear regression analysis results. Only one imaging predictor was used at a time. Model results and significant WMH predictors of cognitive scores adjusted for age at the examination, sex, total years of education, systolic blood pressure and diastolic blood pressure.

|  | Model | | | Parameter estimates | | |
| --- | --- | --- | --- | --- | --- | --- |
| Cognitive test | F | p-value | Adjusted R^2^ | Significant WMH covariate | β (SE) | p-value |
| Letter fluency |  |  |  |  |  |  |
|  | 4.059 | 0.001 | 0.026 | Non T_1_-hypointense deep WMHs | 0.948 (0.434) | 0.029 |
|  |  |  |  |  |  |  |
|  | 5.232 | <0.001 | 0.036 | Non T_1_-hypointense WMH clusters | 1.976 (0.580) | 0.001 |
| Category fluency |  |  |  |  |  |  |
|  | 11.592 | <0.001 | 0.086 | Non T_1_-hypointense deep WMHs | 1.076 (0.520) | 0.039 |
|  |  |  |  |  |  |  |
|  | 12.353 | <0.001 | 0.091 | Non T_1_-hypointense WMH clusters | 2.023 (0.696) | 0.004 |
| Digit span backwards |  |  |  |  |  |  |
|  | 8.346 | <0.001 | 0.061 | Non T_1_-hypointense periventricular WMHs | 1.039 (0.458) | 0.024 |
|  |  |  |  |  |  |  |
|  | 8.227 | <0.001 | 0.060 | Hyperintense *rims* of T_1_-hypointense WMH clusters | 0.916 (0.433) | 0.035 |

Legend: SE, standard error; WMHs, white matter hyperintensities.

Table S4. Bivariate (Pearson) correlations among volumes of sub-classes of WMH, total WMH volume and age.

|  |  | Pearson’s r | p-value |
| --- | --- | --- | --- |
| Non T_1_-hypointense clusters | Deep non T_1_-hypointense WMHs | 0.490 | <0.001 |
|  | T_1_-hypointense clusters | -0.226 | <0.001 |
|  | T_1_-hypointense WMHs | -0.392 | <0.001 |
|  | Total WMHs | -0.166 | <0.001 |
|  | Age | -0.201 | <0.001 |
|  |  |  |  |
| Hyperintense rims | Periventricular non T_1_-hypointense WMHs | 0.905 | <0.001 |
|  | Hypointense cores volume | 0.553 | <0.001 |
|  | T_1_-hypointense clusters | 0.793 | <0.001 |
|  | T_1_-hypointense WMHs | 0.553 | <0.001 |
|  | Total WMHs | 0.806 | <0.001 |
|  | Age | 0.131 | 0.001 |

Legend: WMHs, white matter hyperintensities.
